# Corrigendum

**DOI:** 10.1002/prp2.858

**Published:** 2021-09-01

In Amar et al. (2020), the article^1^ was published with the following errors:

1. In Fig. 3, standard HDL control in Lane 3 was mislabeled as baseline mouse plasma (m1).
2. In Material and Methods section, subsection 2.2, the amount of rhLCAT given to the mice in Figures 6, 7 and SF3 was not clarified.

The correct presentation is listed as below:

1. In Fig. 3, Panel A (left), Filipin and Sudan gels, lane 3 (labeled m1) reflects the migration of purified human HDL standard.
2. In Material and Methods section, subsection 2.2, rhLCAT concentration utilized were 25 mg/kg BW (Fig 6) and 10 mg/kg (Fig 7 and SF3).

The authors would like to apologize for the error.
